# Genomically determined subtypes and clinicopathological features as predictors of the efficacy of preoperative chemotherapy combined with HER2-targeted therapy for early-stage HER2-positive breast cancer

**DOI:** 10.1186/s12885-025-15133-5

**Published:** 2025-11-04

**Authors:** Sayumi Imamura, Kiyoshi Mori, Hiroyuki Yasojima, Makiko Mizutani, Kumiko Okada, Chie Hayashi, Masayuki Mano, Norikazu Masuda

**Affiliations:** 1 Department of Surgery, Breast Oncology, National Hospital Organization Osaka National Hospital, Osaka, Japan; 2 Department of Breast and Endocrine Surgery, Graduate School of Medicine Osaka University, Osaka, Japan; 3 Department of Central Laboratory and Surgical Pathology, National Hospital Organization Osaka National Hospital, Osaka, Japan; 4 Breast Center, Bell Land General Hospital, Osaka, Japan; 5 Division of Breast Surgery, Department of Surgery, Graduate School of Medicine, Kyoto University, 54 Shogoin-Kawahara-cho, Sakyo-ku, Kyoto, 606-8507 Japan

**Keywords:** HER2-positive breast cancer, Neoadjuvant chemotherapy, Intrinsic subtype, 70-gene signature, Multigene assay

## Abstract

**Background:**

Neoadjuvant chemotherapy (NAC) combined with human epidermal growth factor receptor 2 (HER2)-targeted agents is the mainstay of treatment of HER2-positive (HER2 +) breast cancer (BC). We investigated intrinsic subtypes and clinicopathological features as predictors of outcomes of NAC in HER2 + early-stage BC patients.

**Methods:**

Of 186 consecutive patients with early-stage HER2 + BC, as determined by immunohistochemistry (IHC) and fluorescence in situ hybridization, who received NAC plus HER2-targeted agents, 167 with an available biopsy specimen were eligible for inclusion. Intrinsic subtypes were determined by MammaPrint and Blueprint (MP/BP). Whole-slide images of HER2 IHC staining were used to evaluate intensity of HER2 staining.

**Results:**

MP/BP subtype was determined for 124 patients: Luminal-A, 17 patients; Luminal-B, 23 patients; HER2, 76 patients; and Basal, 8 patients. Pathological complete response (pCR) rate was significantly higher in HER2 than in non-HER2 cases (*p* < 0.001). High-intensity (*p* = 0.001) and BP-determined HER2 intrinsic subtype (*p* = 0.024) were identified as independent factors for pCR prediction. Neither Luminal subtype was found in IHC estrogen receptor (ER)-negative patients; however, 54.3% of IHC ER-positive patients had HER2 subtype. Five-year distant disease–free survival by MP/BP subtype was Luminal-A, 92.3%; Luminal-B, 81.6%; HER2, 84.0%; and Basal, 80.0%. Of the 23 patients whose subtypes were compared before and after surgery, 10 had a change in subtype.

**Conclusions:**

High HER2 intensity and HER2 intrinsic subtype could be a means for predicting achievement of pCR. The findings indicate the essential role of MP/BP subtyping in the treatment of HER2 + BC.

**Trial registration:**

UMIN000049957.

## Introduction

Neoadjuvant chemotherapy (NAC) is widely accepted as one of the cornerstones of treatment for breast cancer [1]. The advantage of NAC is that the response to chemotherapy can be evaluated with surgical material, although studies to date have failed to show a clear difference in long-term outcomes between neoadjuvant and adjuvant settings [2]. Pathological complete response (pCR) has been adopted as a surrogate for long-term outcomes in studies of NAC [3]. A meta-analysis performed by Spring et al. showed a significant correlation between pCR and event-free survival or overall survival in NAC, particularly in human epidermal growth factor receptor 2-positive (HER2 +) and triple negative (TN) breast cancer [4]. Although poor surrogacy of pCR was also reported in another meta-analysis of NAC by Conforti et al., they found a strong correlation between pCR and long-term outcomes in early-stage breast cancer [3]. pCR is considered a reasonable surrogate for long-term outcomes of NAC in early-stage HER2 + or TN breast cancer.

In a meta-analysis by Houssami et al., the pCR rate by NAC was reported to be 8.3%, 18.7%, 38.9%, and 31.1% in estrogen receptor–positive (ER +)/HER2-negative (HER2–), ER +/HER2 +, ER-negative (ER–)/HER2 +, and TN breast cancer, respectively, showing a correlation between pCR rate and breast cancer subtype [5]. In recent decades, the pCR rate of NAC continues to increase due to two main driving forces: improved subtyping and the development of HER2-targeted agents. In particular, the development of trastuzumab and pertuzumab has marked a major turning point in HER2 + breast cancer treatment [6]. HER2 + breast cancer is known to be biologically heterogeneous, exhibits various chemosensitivities, and even de-intensification of treatments is suggested necessary for certain subtypes [7].

The MammaPrint^Ⓡ^ (MP) molecular test measures the expression of 70 genes by means of a tissue microarray, produces an index of genomic risk using a proprietary algorithm, and classifies breast cancer into high- and low-risk groups for distant disease [8]. The Blueprint^Ⓡ^ (BP) molecular subtyping test measures the expression of 80 signature genes to classify breast cancer into three subtypes: luminal, HER2, and basal [9]. Combining MP and BP (MP/BP), breast cancer can be divided into four molecular subgroups (MP/BP subtypes): Luminal-A (BP Luminal A for MP low risk), Luminal-B (BP Luminal B for MP high risk), HER2, and Basal.

The present study retrospectively analyzed the effect of NAC used in combination with HER2-targeted agents on each MP/BP subtype of HER2 + (determined by immunohistochemistry, IHC) early-stage breast cancer.

## Methods

### Patient selection

Eligible for the present study were patients who had been diagnosed with HER2 + breast cancer and had received neoadjuvant systemic therapy including HER2-targeted agents, followed by definitive surgery, at the National Hospital Organization Osaka National Hospital, Osaka, Japan, between May 2005 and July 2021, and for whom formalin-fixed, paraffin-embedded (FFPE) blocks of pretreatment biopsy or surgical specimens were available from the hospital archives.

### Data collection and processing

Information, including age, tumor size, clinical stage, ER status, progesterone receptor (PgR) status, and HER2 staining rate, Ki-67 level, Bloom and Richardson grade, and HER2/chromosome enumeration probe 17 (CEP17) ratio in HER2 IHC 2 + cases, was collected from pathology reports and patient charts. ER and PgR status were considered positive if there was at least 1% positive staining by IHC. ER positivity between > 1% and < 10% was separately defined as ER weakly positive. HER2 status was judged positive if staining intensity was IHC 3 + or IHC 2 + with HER2 gene amplification by fluorescence in situ hybridization (FISH). pCR was defined as ypT0N0 or ypTisN0. Ki-67 level was evaluated, using a 20% cutoff.

### Analysis of HER2 staining intensity and heterogeneity

**Fig. 1:**
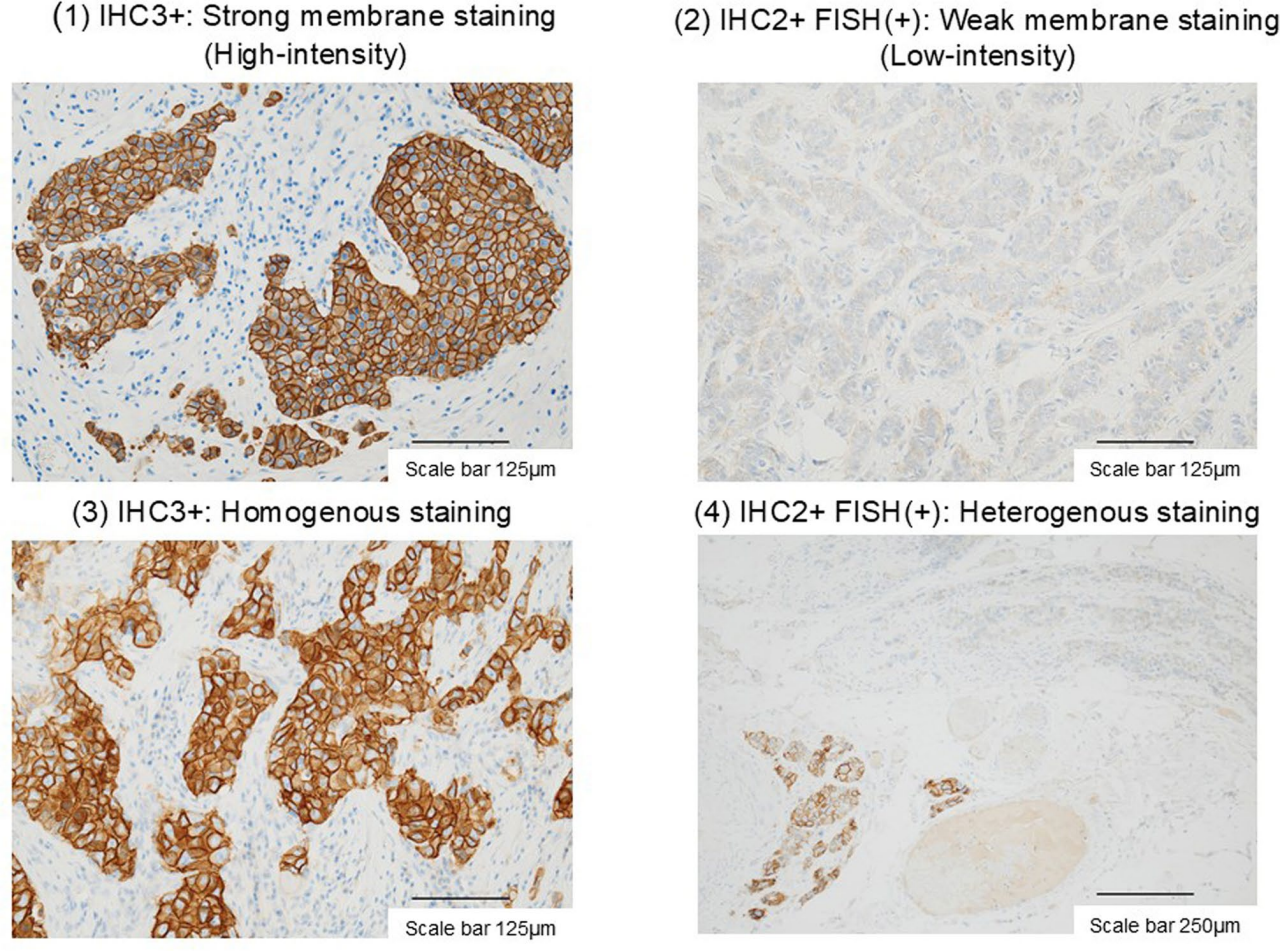
Typical examples of immunohistochemistry (IHC) human epidermal growth factor receptor 2 (HER2) staining patterns. FISH, fluorescence in situ hybridization

This analysis was carried out using biopsy specimens obtained from 167 patients and stored at our hospital. After being subjected to HER2 immunohistochemical staining, slides prepared using these specimens were first examined under a light microscope before being digitized for image analysis. HER2 staining patterns were evaluated according to the intensity and heterogeneity of staining (Fig. 1). Staining intensity was quantified using ImageJ software (0 = white, 225 = black) and defined as High-intensity or Low-intensity based on its being, respectively, higher or lower than the cutoff value determined using the Youden index, which maximized the area under the receiver operating characteristic (ROC) curve. HER2 staining was classified as “heterogeneous” when areas of intense staining were interspersed with faint staining, or “homogeneous” when membrane staining was uniform.

### Analysis of genomically determined subtypes

For MP/BP analysis (performed by Agendia Inc., Amsterdam, the Netherlands), 10 consecutive slides (5 μm each) were prepared from FFPE tissue blocks of biopsy specimens and surgical specimens. Biopsy specimens were submitted to MP/BP for all cases in which FFPE blocks were stored at our hospital. Surgical specimens were submitted if more than one-third of the tumor volume remained after NAC. RNA was extracted from thinly sliced samples, and the extracts were subjected to quality control prior to sequencing analysis. Samples deemed by quality control to be of insufficient RNA quality were excluded from the analysis.

The primary endpoints of this study are differences in pCR rates based on staining intensity and heterogeneity, as well as pCR rates by each intrinsic subtype. Secondary endpoints were defined as postoperative distant disease-free survival (D-DFS) rates for each intrinsic subtype. D-DFS was defined in accordance with the guidelines regarding clinical events to be included, namely death from any cause and distant recurrence [10].

### Statistical analysis

All statistical analyses were performed using R version 4.2.0 (R Project for Statistical Computing, Vienna, Austria). Univariate analyses were performed using the chi-squared test or Fisher’s exact test for 2 × 2 tables, and the chi-squared test for n × 2 tables (*n* ≥ 3). Variables with *p* < 0.05 were entered into a multivariate logistic regression model for pCR, or a Cox proportional hazards model for D-DFS. Because of the small number of patients with each subtype, certain analyses, such as the comparison of survival outcomes, were performed for HER2 vs. non-HER2. The reported statistical significance levels were all two-sided, and *p*-values of less than 0.05 were considered statistically significant.

## Results

### Clinical features

**Fig. 2:**
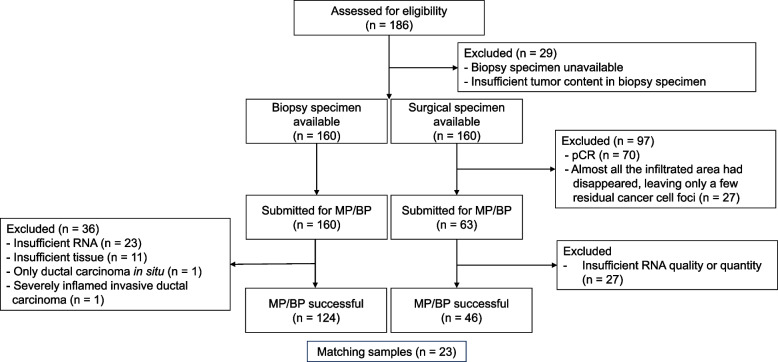
Disposition of the patients included in the present study. Histopathological results for 23 patients were available for comparison of biopsy specimens with matched surgical samples, after 40 patients had been excluded for the following reasons: MammaPrint and Blueprint (MP/BP) molecular type tests had not been performed on the biopsy specimens (*n* = 9), results for biopsy specimens were not detected (*n* = 10), and results for surgical specimens were not detected (*n* = 21). pCR, pathological complete response

MP/BP was successfully performed on biopsy specimens from 124 of the 160 patients who met the inclusion criteria for the present study (Fig. 2). Table 1 summarizes the characteristics of these 124 patients and the clinicopathological features of their tumors. The median age of the patients was 56.0 years, and 65% were aged ≥ 50 years. All had received taxane-containing NAC with trastuzumab, and additional anthracycline-containing chemotherapy had been administered in 67.0% of cases. The combination of trastuzumab and pertuzumab was used for only 24 patients (19.4%), since pertuzumab in neoadjuvant settings had become insurable in Japan only in October 2018.

**Table 1:** Characteristics of the patients for whom MammaPrint and Blueprint subtyping was successfully performed in the present study, and the clinicopathological features of their tumors

Characteristic	pCR (*n* = 51)	non-pCR (*n* = 73)	Univariate analysis			Multivariate analysis		
			Odds ratio	95% CI	*p*-value	Odds ratio	95% CI	*p*-value
Age, years								
≤ 50	16	25	0.88	0.41–1.88	0.847^a^			
> 50	35	48						
cT								
T1	10	19	0.69	0.29–1.65	0.518^a^			
T2, T3, T4	41	54						
cN								
N0	36	53	0.91	0.41–2.00	0.814^a^			
N1, N2, N3	15	20						
ER								
Negative	22	12	4.03	1.87–8.68	< 0.001^a^	3.23	1.31–7.99	0.011
Weakly positive	7	6						
Positive	22	55						
HER2								
IHC 3 +	49	64	3.45	0.71–16.67	0.193^b^			
IHC 2 + and FISH +	2	9						
Histological grading								
HG 3	40	41	2.84	1.26–6.39	0.012^b^	3.12	1.18–8.22	0.021
HG 1,2	11	32						
Ki-67^c^								
< 20%	3	12	0.36	0.10–0.38	0.109^b^			
≥ 20%	37	54						
Unknown	11	7						
Neoadjuvant chemotherapy								
Taxane + anthracycline	32	51	0.73	0.00–1.37	0.442^a^			
Taxane only	19	22						
Anti-HER2 therapy								
Trastuzumab alone	40	60	0.79	0.32–1.93	0.648^a^			
Trastuzumab + pertuzumab	11	13						
HER2 staining intensity								
High intensity	40	25	6.98	3.06–15.92	< 0.001^a^	4.12	1.55–10.90	0.004
Low intensity	11	48						
Intrinsic subtype^d^								
Luminal-A	0	17	10.16	3.83–26.81	< 0.001^a^	4.47	1.46–13.70	0.009
Luminal-B	2	21						
HER2	45	31						
Basal	4	4						

*ER* estrogen receptor, *FISH* fluorescence in situ hybridization*, HER2* human epidermal growth factor receptor 2, *HG *histological grade, *IHC* immunohistochemistry

^a^Fisher’s exact test; *p*-values for each patient subgroup have been corrected using the Hochberg’s method

^b^Chi-square test; *p*-values for each patient subgroup have been corrected using the Hochberg’s method

^c^The chi-square test for Ki-67 was performed for patients excluding those with unknown Ki-67 status

^d^Because of the small number of patients in each group, the intrinsic subtypes were divided into two groups (HER2 and others) and statistically examined

Reasons for the failure of MP/BP subtyping in the remaining 36 of the 160 biopsy specimens submitted were insufficient RNA (*n* = 23), insufficient tissue (*n* = 11), ductal carcinoma in situ only (*n* = 1), or severe inflammation (*n* = 1). Among the 124 cases for which MP/BP subtype was determined, the distribution of subtypes was as follows: Luminal-A, 17; Luminal-B, 23; HER2, 76; and Basal, 8. Of the 92 cases identified by IHC as ER +, more than half were non-Luminal (Luminal-A, 17 cases [18.5%]; Luminal-B, 23 cases [25.0%]; HER2, 50 cases [54.3%]; Basal, 2 cases [2.2%]). All 32 ER– cases were non-Luminal (HER2, 26 cases [81.2%]; Basal, 6 cases [18.8%]). All Basal cases, and all except 2 HER2 cases, were classified as MP high risk.

### Treatment response according to the MP/BP system

Of the 124 patients whose biopsy specimens were successfully subtyped by MP/BP, 51 (41.1%) achieved pCR. pCR rate was low in those with the Luminal-A (0%) and Luminal-B (8.6%) subtypes, but high in those with the HER2 (59.2%) and Basal (50.0%) subtypes. Thus, pCR rate was higher in HER2 cases than in non-HER2 cases (59.2% vs. 12.5%; odds ratio, OR, 10.16 [95% confidence interval, CI, 3.58–26.81], *p* < 0.001), and higher in IHC ER– cases than in IHC ER + cases within the intrinsic HER2 subtype (73.0% vs. 50.0%; OR, 2.92 [95% CI, 1.06–8.09], *p* = 0.03). In ER-positive cases, BP classification correlated well with pCR rate; even in the IHC ER + cases, 50% of patients with BP HER2 achieved pCR, which was a significantly higher proportion than for patients with IHC ER + BP non-HER2 tumors (5.13%) (OR, 19.50 [95% CI, 4.11–92.50], *p* < 0.01).

### HER2 staining pattern and treatment response

HER2 staining intensity was evaluated for 167 biopsy specimens with available whole-slide images. ROC analysis identified 149.50 as the optimal cutoff value. Mean intensity score was 172.6 in the High-intensity group (*n* = 87) and 111.9 in the Low-intensity group (*n* = 80). For the biopsy specimens from 124 patients for whom MP/BP results were available, 65 were of High-intensity and 59 were of Low-intensity. The pCR rates for the High-intensity and Low-intensity groups were 61.5% and 18.6%, respectively. High-intensity was strongly associated with the intrinsic HER2 subtype. When combining data for HER2 IHC intensity and intrinsic subtype, four groups were identified: High-intensity HER2 (pCR rate, 69.1%), High-intensity non-HER2 (pCR rate, 20.0%), Low-intensity HER2 (pCR rate, 33.3%), and Low-intensity non-HER2 (pCR rate, 10.5%) (Table 2).

**Table 2:** Comparison of pCR rates by HER2 staining intensity and intrinsic subtypes in biopsy specimens from 124 patients with available MammaPrint and Blueprint results

	HER2 intensity		Intrinsic subtype based on HER2 status		
	*n*	pCR, *n* (%)		*n*	pCR, *n* (%)
High-intensity	65	40 (61.5)	HER2	55	38 (69.1)
			Others	10	2 (20.0)
Low-intensity	59	11 (18.6)	HER2	21	7 (33.3)
			Others	38	4 (10.5)
Total	124	51 (41.1)	HER2	76	45 (59.2)
			Others	48	6 (12.5)

*HER2* human epidermal growth factor receptor 2, *pCR* pathological complete response

Heterogeneous HER2 staining was observed in 4 cases, all of which were classified as High-intensity. About half of the tumor remained after NAC in 2 cases, and about one-third of infiltrating cancer tissue remained after NAC in the other 2 cases.

### Factors predicting pCR achievement

The results of univariate analyses showed negative or weakly positive ER status, high Bloom and Richardson histological grade, HER2 intrinsic subtype, and High-intensity of HER2 staining to be significantly correlated with achievement of pCR. Multivariate logistic regression analysis of data for factors identified as significant in univariate analysis (i.e. ER status, histological grade, HER2 staining intensity, and intrinsic subtype) showed all four to be independent predictors of pCR achievement (Table 1).

### Prognosis

**Fig. 3:**
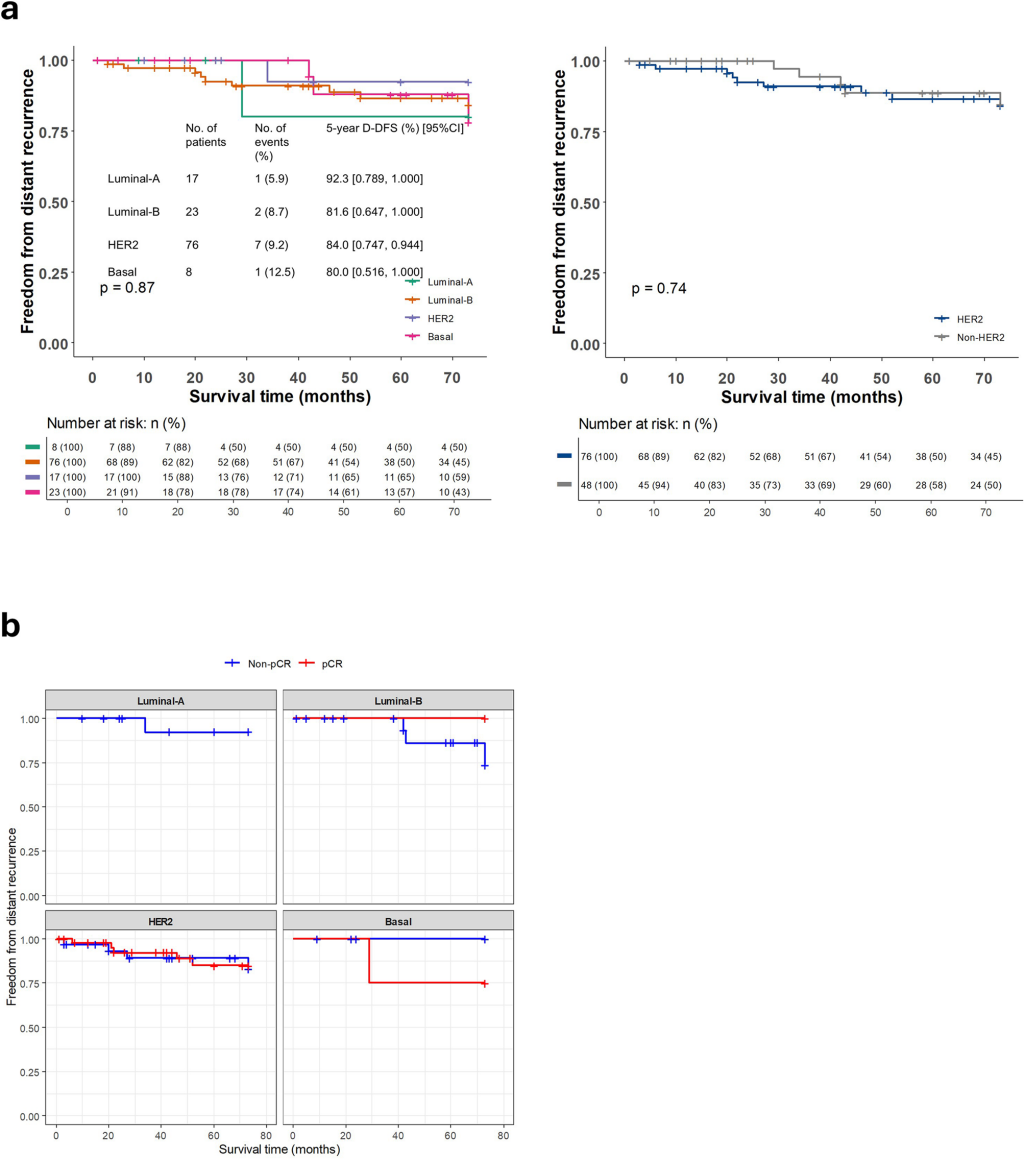
Kaplan–Meier estimates of 5-year distant disease-free survival (D-DFS) for the 124 patients included in the present study: **a **comparison of results for patients with the Luminal-A, Luminal-B, HER2, and basal subtypes, and of results for those in the non-HER2 vs. HER2 groups; **b **comparison of results for patients with each subtype (i.e. Luminal-A, Luminal-B, HER2, and Basal), based on whether they did or did not achieve pathological complete response (pCR) (i.e. pCR vs. non-pCR). CI, confidence interval; HER2, human epidermal growth factor receptor 2

The median follow-up period was 73.0 months, ranging from 5.7 to 154.0 months. Overall, 13 (10.5%) of the 124 patients for whom MP/BP results were available experienced either local recurrence (2 patients, 1.6%) and/or distant disease (11 patients, 8.9%). In the group with distant recurrence, deaths due to breast cancer were recorded during the observation period, but no deaths due to causes other than breast cancer were confirmed. Kaplan–Meier estimates of 5-year D-DFS stratified by intrinsic subtype were 92.3% (95% CI, 0.789–1.000) for Luminal-A, 81.6% (0.647–1.000) for Luminal-B, 84.0% (95% CI, 0.747–0.944) for HER2, and 80.0% (95% CI, 0.516–1.000) for Basal (Fig. 3a). Cox proportional hazards analysis showed no significant differences between MP/BP subtypes. Kaplan–Meier curves for the pCR group and the non-pCR group according to intrinsic subtype are shown in Fig. 3b. No patients with the Luminal-A subtype achieved pCR. Regarding the other subtypes (Luminal-B, HER2, and Basal), there were no differences in prognosis between the pCR and non-pCR groups.

**Fig. 4:**
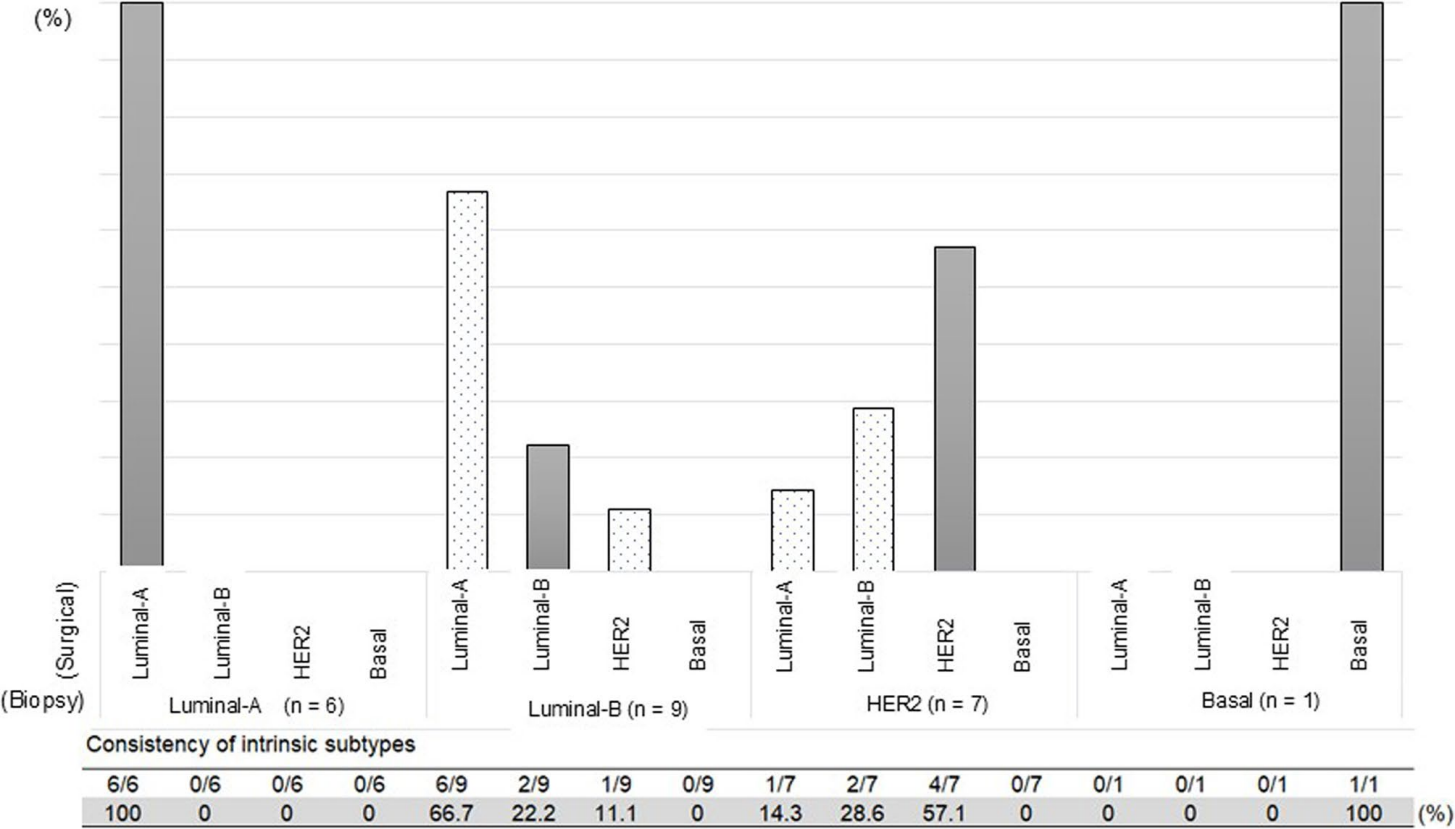
Intrinsic subtype changes attributable to neoadjuvant chemotherapy: comparison of results for the biopsy specimens and matched surgical samples of 23 patients. The subtype changed for 10 patients. HER2, human epidermal growth factor receptor 2

Surgical samples with approximately one-third of all cancer cells remaining after NAC were eligible for MP/BP testing. However, for 24 of these, testing was not possible, due to insufficient RNA quantity; they were obtained mainly from tumors that were small originally (cT1–T2). Regarding the biopsy specimens, the intrinsic subtype was either unexamined or not determined in 19 cases. Having excluded these surgical samples and biopsy specimens, it was possible to compare in each of 23 cases the intrinsic subtype determined using a biopsy specimen with that determined using a surgical sample. The latter differed from the former in 10 of these 23 cases (Fig. 4).

Recurrence occurred in one of the 23 patients whose cancer had been subtyped using both a biopsy specimen and a surgical sample. For that patient, in both cases the subtype was Luminal-B.

## Discussion

The present study showed that negative or weakly positive ER status, histological grade 3, MP/BP HER2 subtype, and High-intensity of HER2 IHC staining were independent factors significantly predicting achievement of pCR with NAC. It is noteworthy that the pCR rates were considerably different among MP/BP subtypes, even in IHC/FISH HER2 + breast cancer. These results are consistent with previous reports. Choi et al. showed that the pCR rate by NAC combined with trastuzumab was significantly higher in the cases with high HER2 gene amplification in FISH (> 10.0 HER2 gene copies per cell) than those of low amplification (6–10 HER2 gene copies per cell) (56% vs. 22%; *p* < 0.005) [11]. Another study also demonstrated a positive correlation between FISH amplification of HER2 and the pCR rate by NAC combined with trastuzumab [12]. Other previous studies revealed that high amplification or high mRNA expression of HER2 showed a positive correlation with response to trastuzumab in a subset of patients with IHC/FISH HER2 + breast cancer [13, 14]. However, in these studies, HER2 was semi-quantified by a single genomic test. In the present study, a multigene assay using MP/BP was performed, and hence subtyping was more thorough and comprehensive.

In the present study, we also evaluated whether there was heterogeneity in IHC staining in biopsy specimens, but only 4 out of 167 cases showed obvious heterogeneity. Of these 4 cases, 2 were pCR and 2 were non-pCR, but due to the small number of cases, we were unable to sufficiently evaluate the impact of IHC heterogeneity on treatment efficacy. Differences in treatment efficacy based on FISH copy number have been reported, and it has been suggested that cases with strong heterogeneity may not respond adequately to HER2-targeted agents [15]. However, there are few studies that evaluate HER2 heterogeneity using IHC alone and examine the efficacy of drug therapy, so it may be that IHC alone is insufficient for evaluating HER2 heterogeneity.

The pCR rates ranged from 0 in Luminal-A patients to 59.2% in HER2 patients, depending on intrinsic subtype. HER2 patients with IHC/FISH HER2 + breast cancer had significantly better results than non-HER2 patients, especially those with the BP luminal subtype, in response to NAC combined with HER2-targeted agents. These results were consistent with the findings from the PAMELA study, in which PAM50 was used as a multigene assay to define genomic subtypes so as to identify patients who are likely to benefit from dual HER2 blockade with lapatinib and trastuzumab as NAC without cytotoxic agents [16]. About half of IHC/FISH ER + HER2 + breast cancer patients were in the Luminal subgroup and had a significantly lower pCR rate but a better prognosis, even without having achieved pCR in response to NAC. Whereas NAC combined with HER2-targeted agents is considered suitable treatment for cases of HER2 breast cancer, it appears that NAC regimens should be reconsidered for the treatment of non-HER2 cases of IHC/FISH HER2 + breast cancer. NAC combined with HER2-targeted agents might be an overtreatment for Luminal cases. Indeed, based on the results of the KATHERINE study [17], it is common practice for trastuzumab emtansine (T-DM1) to be administered to prevent distant recurrence in IHC HER2-positive patients who have failed to achieve pCR with NAC. However, our study suggests that patients with intrinsic Luminal tumors are less likely to relapse even if pCR was not achieved, so administration of additional potent anti-cancer might be excessive for them. In the present study, the pCR rate was slightly high in patients who received NAC in combination with trastuzumab and pertuzumab, compared with those who received NAC with trastuzumab alone. Although this finding is not based on a strict comparison, similar results have been reported in previous global clinical trials such as the NeoSphere trial [18], and the TRYPHAENA trial [19].

In the HERA study, FISH amplification and HER2 staining intensity were positively correlated [20]. We also found a good correlation between High-intensity and HER2 status, together with a highly significant positive correlation between HER2 staining intensity and pCR. These results suggest that a high intensity of HER2 IHC staining can be an alternative to HER2 in predicting achievement of pCR when genomic analysis is unavailable. In the absence of homogeneous membranous staining, attention should be paid, as the therapeutic effect might be limited even with high staining intensity.

Given that in the present study the observation period was relatively short (73.0 months), the number of patients who experienced postoperative recurrence was low (local recurrence, 1.6%; distant recurrence, 8.9%). Among patients who experienced distant recurrence, there were cases of death due to breast cancer, but no cases of death due to causes other than breast cancer were observed. The 5-year D-DFS rate was 92.3% for patients with the Luminal-A subtype, 81.6% for those with the Luminal-B subtype, 84.0% for those with the HER2 subtype, and 80.0% for those with the Basal subtype. No significant differences in prognosis were observed among the intrinsic subtypes. Similarly, no differences in prognosis were observed when tumors were classified as HER2 or non-HER2. Although differences in pCR rates were observed for each intrinsic subtype, the lack of differences in distant recurrence was considered due to the relatively short observation period and the small number of recurrence cases. In addition, in Japan, T-DM1 was approved only from August 2020, for the purposes of health insurance coverage, as adjuvant therapy for early-stage HER2-positive breast cancer patients who have not achieved pCR. In the present study, only 6 of 73 patients in the non-pCR group received T-DM1, and we consider it important to note this difference from current clinical practice.

In the present study, the results of MP/BP could not be obtained for 36 (22.5%) of all 160 biopsy specimens, mainly because of insufficient RNA quality or quantity. The quality of RNA was insufficient in 5.8% of 86 samples collected between 2012 and 2021, compared with 24.3% of the 74 samples collected between 2005 and 2011. The difference was statistically significant (*p* < 0.001). Tumor tissue older than 10-years might be unsuitable for analysis using MP/BP. The key limitations of this study are that it is retrospective study, with a limited number of patients, and the chemotherapy regimens and HER2-targeted therapy varied with patient background and the era. Moreover, it is well known that tumors of the Luminal-A type tend to recur late, often more than 5 years after surgery. Considering the relatively short observation period in the present study, the prognosis for Luminal-A type tumors has not been sufficiently evaluated.

## Conclusions

Our study highlights highly significant correlations between HER2 staining intensity and intrinsic subtypes, and both were significant predictors of therapeutic effects of NAC combined with HER-targeted agents. The findings indicate the essential role of MP/BP subtyping in the treatment of HER2 + breast cancer. Although intrinsic HER2 showed a higher pCR rate, about half of IHC ER + HER2 + breast cancers were of the Luminal subtype, with a better prognosis. Further analysis is needed on the treatment strategies for intrinsic Luminal-subtype breast cancers with positive HER2 IHC staining.

## Data Availability

The original data are not publicly available due to privacy/ethical restrictions, but are available from the corresponding author on reasonable request.

## Abbreviations

BP: Blueprint^Ⓡ^
CEP: Chromosome enumeration probe
CI: Confidence interval
D-DFS: Distant disease-free survival
ER: Estrogen receptor
FFPE: Formalin-fixed, paraffin-embedded
FISH: Fluorescence in situ hybridization
HER2: Human epidermal growth factor receptor 2
IHC: Immunohistochemistry
MP: MammaPrint^Ⓡ^
NAC: Neoadjuvant chemotherapy
OR: Odds ratio
pCR: Pathological complete response
PgR: Progesterone receptor
RNA: Ribonucleic acid
ROC: Receiver operating characteristic
T-DM1: Trastuzumab emtansine
TN: Triple negative

## References

[1] Wolmark N, Wang J, Mamounas E, Bryant J, Fisher B. Preoperative chemotherapy in patients with operable breast cancer: nine-year results from National Surgical Adjuvant Breast and Bowel Project B-18. J Natl Cancer Inst Monogr. 2001;30:96–102. 10.1093/oxfordjournals.jncimonographs.a003469. 10.1093/oxfordjournals.jncimonographs.a003469 11773300

[2] Mauri D, Pavlidis N, Ioannidis JPA. Neoadjuvant versus adjuvant systemic treatment in breast cancer: a meta-analysis. J Natl Cancer Inst. 2005;97:188–94. 10.1093/jnci/dji021. 15687361 10.1093/jnci/dji021

[3] Conforti F, Pala L, Sala I, Oriecuia C, De Pas T, Specchia C, et al. Evaluation of pathological complete response as surrogate endpoint in neoadjuvant randomized clinical trials of early stage breast cancer: systematic review and meta-analysis. BMJ. 2021;375:e066381. 10.1136/bmj-2021-066381. 34933868 10.1136/bmj-2021-066381 PMC8689398

[4] Spring LM, Fell G, Arfe A, Sharma C, Greenup R, Reynolds KL, et al. Pathologic complete response after neoadjuvant chemotherapy and impact on breast cancer recurrence and survival: a comprehensive meta-analysis. Clin Cancer Res. 2020;26:2838–48. 10.1158/1078-0432.CCR-19-3492. 32046998 10.1158/1078-0432.CCR-19-3492 PMC7299787

[5] Houssami N, Macaskill P, von Minckwitz G, Marinovich ML, Mamounas E. Meta-analysis of the association of breast cancer subtype and pathologic complete response to neoadjuvant chemotherapy. Eur J Cancer. 2012;48:3342–54. 10.1016/j.ejca.2012.05.023. 22766518 10.1016/j.ejca.2012.05.023

[6] Kreutzfeldt J, Rozeboom B, Dey N, De P. The trastuzumab era: current and upcoming targeted HER2+ breast cancer therapies. Am J Cancer Res. 2020;10:1045–67. 32368385 PMC7191090

[7] Hamilton E, Shastry M, Schiller SM, Ren R. Targeting HER2 heterogeneity in breast cancer. Cancer Treat Rev. 2021;100:102286. 10.1016/j.ctrv.2021.102286. 34534820 10.1016/j.ctrv.2021.102286

[8] Cardoso F, van’t Veer LJ, Bogaerts J, Slaets L, Viale G, Delaloge S, et al. 70-gene signature as an aid to treatment decisions in early-stage breast cancer. N Engl J Med. 2016;375:717–29. 10.1056/NEJMoa1602253. 27557300 10.1056/NEJMoa1602253

[9] Whitworth P, Stork-Sloots L, de Snoo FA, Richards P, Rotkis M, Beatty J, et al. Chemosensitivity predicted by blueprint 80-gene functional subtype and MammaPrint in the prospective neoadjuvant breast registry symphony trial (NBRST). Ann Surg Oncol. 2014;21:3261–7. 10.1245/s10434-014-3908-y. 25099655 10.1245/s10434-014-3908-y PMC4161926

[10] Gourgou-Bourgate S, Cameron D, Poortmans P, Asselain B, Azria D, Cardoso F, et al. Guidelines for time-to-event and point definitions in breast cancer trials results of the DATECAN initiative (*d*efinition for the *a*ssessment of *t*ime-to-event *e*ndpoints in *can*cer trials). Ann Oncol. 2015;26(5):873–9. 10.1093/annonc/mdv106. 25725046 10.1093/annonc/mdv106

[11] Choi JH, Jeon CW, Kim YO, Jung S. Pathological complete response to neoadjuvant trastuzumab and pertuzumab therapy is related to human epidermal growth factor receptor 2 (HER2) amplification level in HER2-amplified breast cancer. Medicine (Baltimore). 2020;99:e23053. 10.1097/MD.0000000000023053. 33181670 10.1097/MD.0000000000023053 PMC7668516

[12] Arnould L, Arveux P, Couturier J, Gelly-Marty M, Loustalot C, Ettore F, et al. Pathologic complete response to trastuzumab-based neoadjuvant chemotherapy is related to the level of HER-2 amplification. Clin Cancer Res. 2007;13:6404–9. 17975153 10.1158/1078-0432.CCR-06-3022

[13] Fuchs EM, Köstler WJ, Horvat R, Hudelist G, Kubista E, Attems J, et al. High-level ERBB2 gene amplification is associated with a particularly short time-to metastasis, but results in a ahigh rate of complete response once trastuzumab-based therapy is offered in the metastatic setting. Int J Cancer. 2013;135:224–31. 10.1002/ijc.28660. 10.1002/ijc.28660 24311197

[14] Giuliani R, Durbecq V, Di Leo A, Paesmans M, Larsimont D, Leroy JY, et al. Phosphorylated HER-2 tyrosine kinase and HER2/neu gene amplification as predictive factors of response to trastuzumab in patients with HER-2 overexpressing metastatic breast cancer (MBC). Eur J Cancer. 2007;43:725–35. 10.1016/j.ejca.2006.11.019. 17251007 10.1016/j.ejca.2006.11.019

[15] Otto Metzger G, Giuseppe V, Shayna S, Lorenzo T, Denise AY, Ingrid AM, et al. Impactof HER2 heterogeneity on treatment response of early-stage HER2-positive breast cancer : phase II neoadjuvant clinical trial of T-DM1 combined with pertuzumab. Cancer Discov. 2021;11(10):2474–87. 10.1158/2159-8290.CD-20-1557. 33941592 10.1158/2159-8290.CD-20-1557 PMC8598376

[16] Llombart-Cussac A, Cortes J, Pare L, Galván P, Bermejo B, Martínez N, et al. HER2-enriched subtype as a predictor of pathological complete response following trastuzumab and lapatinib without chemotherapy in early-stage HER2-positive breast cancer (PAMELA): an open-label, single-group, multicentre, phase 2 trial. Lancet Oncol. 2017;18:545–54. 10.1016/S1470-2045(17)30021-9. 28238593 10.1016/S1470-2045(17)30021-9

[17] von Minckwitz G, Huang CS, Mano MS, Loibl S, Mamounas EP, Untch M, et al. Trastuzumab emtansine for residual invasive HER2-positive breast cancer. N Engl J Med. 2019;380:617–28. 10.1056/NEJMoa1814017. 30516102 10.1056/NEJMoa1814017

[18] Gianni L, Pienkowski T, Im Y, Tseng LM, Liu MC, Lluch A, et al. 5-year analysis of neoadjuvant pertuzumab and trastuzumab in patients with locally advanced, inflammatory, or early-stage HER2-positive breast cancer (NeoSphere): a multicentre, open-label, phase 2 randomised trial. Lancet Oncol. 2016;17:791–800. 10.1016/S1470-2045(16)00163-7. 27179402 10.1016/S1470-2045(16)00163-7

[19] Schneeweiss A, Chia S, Hickish T, Harvey V, Eniu A, Hegg R, et al. Pertuzumab plus trastuzumab in combination with standard neoadjuvant anthracycline-containing and anthracycline-free chemotherapy regimens in patients with HER2-positive early breast cancer: a randomized phase II cardiac safety study (TRYPHAENA). Ann Oncol. 2013;24:2278–84. 10.1093/annonc/mdt182. 23704196 10.1093/annonc/mdt182

[20] Piccart-Gebhart MJ, Procter M, Leyland-Jones B, Goldhirsch A, Untch M, Smith I, et al. Trastuzumab after adjuvant chemotherapy in HER2-positive breast cancer. N Engl J Med. 2005;353:1659–72. 10.1056/NEJMoa052306. 16236737 10.1056/NEJMoa052306

